# An Improved Pearson's Correlation Proximity-Based Hierarchical Clustering for Mining Biological Association between Genes

**DOI:** 10.1155/2014/357873

**Published:** 2014-06-16

**Authors:** P. M. Booma, S. Prabhakaran, R. Dhanalakshmi

**Affiliations:** ^1^Department of Computer and Engineering, KCG College of Technology, KCG Nagar, Rajiv Gandhi Salai, Karapakkam, Chennai, Tamil Nadu 600097, India; ^2^Department of Computer Science and Engineering, SRM University, SRM Nagar, Kattankulathur, Kanchipuram, National Highway 45, Potheri, Tamil Nadu 603203, India

## Abstract

Microarray gene expression datasets has concerned great awareness among molecular biologist, statisticians, and computer scientists. Data mining that extracts the hidden and usual information from datasets fails to identify the most significant biological associations between genes. A search made with heuristic for standard biological process measures only the gene expression level, threshold, and response time. Heuristic search identifies and mines the best biological solution, but the association process was not efficiently addressed. To monitor higher rate of expression levels between genes, a hierarchical clustering model was proposed, where the biological association between genes is measured simultaneously using proximity measure of improved Pearson's correlation (PCPHC). Additionally, the Seed Augment algorithm adopts average linkage methods on rows and columns in order to expand a seed PCPHC model into a maximal global PCPHC (GL-PCPHC) model and to identify association between the clusters. Moreover, a GL-PCPHC applies pattern growing method to mine the PCPHC patterns. Compared to existing gene expression analysis, the PCPHC model achieves better performance. Experimental evaluations are conducted for GL-PCPHC model with standard benchmark gene expression datasets extracted from UCI repository and GenBank database in terms of execution time, size of pattern, significance level, biological association efficiency, and pattern quality.

## 1. Introduction

The adoption of microarray technology provides the biologists to be competent at monitoring thousands of genes expression in a solitary experiment on a small chip with the existence of several microarray gene expression datasets that openly exist on the Internet. The dataset in it comprises huge count of gene expression values and emphasizes a precise method to differentiate between the knowledge and useful information from these microarray gene expression datasets. The application of support vector machines (SVM) has been shown to be of superior performance during the analysis of microarray gene expression data when compared to the other classification algorithms such as decision trees and linear discrimination. The top ranked informative genes using a filtering algorithm in [1] with respect to structural risk minimization used the support vector machine. But the SVM as the base classifier failed in dealing with overfitting problems owed to the boosting approach.

On the other hand, the DNA microarray technology evaluates the expression level of thousands of genes at the same time. With this evaluation, certain genes could be related to an exact type of cancer, whereas many of them consist of irrelevant or redundant features that result in a high influential factor on the speed and accuracy of classification. Developing predictive and prognostic classifiers to recognize the patient highly requires action and forms as the most excellent candidate form for specific treatments. As microarrays construct as much of data from every specimen, [2] the method provides with the greater opportunity for discovering huge dangers on misleading claims. DNA microarrays provide enormous occasion for discovery and progress of predictive oncology but with a greater tradeoff value between the opportunity and mounting false claims.

Moreover, the connections between morphometry and molecular characterization in the TCGA and REM BRANDT datasets as shown in [3] failed in investigating the morphology of blood vessels within the context of tumor progression. The main focus was concentrated on the portion of pathology image investigation and illustrates the challenges associated with analyzing and integrating large-scale image datasets with molecular characterizations.

Based on the gene expression profiles, the selection of gene with the recognition of the optimal subset of relevant genes is one of the foremost challenges in cancer classification. Genomic regulation forecast single-peaked distributions method of expression value decays according to power laws in [4] with the feature exponent inversely associated with the product of the connectivity of the network times the regulatory strength of bound transcription factors. Information on structural properties and on the interactions of regulatory elements was at the same time used to build up a framework of basic characteristics of expression spectra. It helped in progressing of classification accuracy, minimizing the computational cost, and maximizing the insight into the inherent cancer mechanisms. SC3 [5], the author presented spectral clustering for performing clustering on the gene and cancer sample dimension and finally partitioned the consensus matrix from multiple clustering solutions. But the flaw was that this method was suitable for only cancer gene expression profiles.

Kidney paired donation (KPD) programs as presented in [6] provide an innovative approach for increasing the number of accessible kidneys. But KPD failed to focus on the incorporation of additional KPD allocation algorithms. Also, KPD failed to develop modeling of certain significant system parameters as functions of practical donor or candidate characteristics and evaluate the differences and relationships. CMOS integrated circuit technology is leveraged for biotechnology applications as presented in [7], in the form of affinity-based assays, in which the conventional passive solid supports were restored by active integrated circuit chips. Direct use of the chip as a solid support allowed further complicated task on surface chemistry. In [8], the author designed an evolutionary algorithm (EA) to evolve top-scoring pairs called EvoTSP that allowed identifying the more advanced gene relations. The major variants of relative expression algorithms were balanced using EA by introducing weights to the top-scoring pairs. A differential network-based framework [9] was designed to detect the cancer-related genes using boosting regression method based on likelihood score. But the biological data applied to the framework was limited which remained an open issue to be solved.

Gene expression was caused by O_3_ in [10], with the combination of CO_2_. O_3_ induced new type of reaction that oxidative pressure and previous leaf senescence, seen as decrease appearance of photosynthesis and carbon fixation-related genes, increases expression of senescence-associated genes. Biclustering technique is capable of detecting positively and unenthusiastically coregulated genes in [11]. These biclustering technique measures MSR, Euclidean proximity, and also correlation using pattern-based approach for identifying the comparison between the genes. Moreover, it deterministically identifies all possible biclusters using a nongreedy method in addition to the application of the polynomial time.

The methods involved in gene selection are classified into filter and wrapper approaches. The filter approach of gene selection selects the top ranked genes on the basis of their individual discriminative power without concentrating on and including any induction algorithm. Also, genes are evaluated using different types of common individuality measures in data, and the performance of filter-based gene selection is measured efficiently. It is highly advantageous for high-dimensional data outstanding to its linear time complexity, but it cannot find out the synergy result or suppressible in the middle of genes. The wrapper method, in contrast, assesses applicant gene subsets using an induction algorithm. Since the predictive accuracy of the induction algorithm determines the integrity of the selected subsets, it is competent to allow correlations among genes but is often computationally expensive.

The temporal and spatial correlations and the reliability in the trajectory of datasets of moving objects as shown in [12] are repeatedly modelled as sequential patterns for use in data mining. Distributed mining algorithm is comprised of local group movement pattern mining (GMPMine) algorithm that removes local group information and cluster ensembling (CE) algorithm that merges and improves the local grouping results. An automated approach for activity tracking as illustrated in [13] identified frequent activities that naturally occur in an individual's routine. But the automated approach fails to automatically select the number based on the resident's lifestyle. Seeding the clusters based on smart environment information incrementally modifies the patterns, clusters, and models as activities alter in excess of time.

Method for trajectory segmentation and sampling based on the representativeness of the (sub)trajectories in the MOD as shown in [14] failed to hold each subtrajectory of the sampling set by different subtrajectories. The most representative subtrajectories under the minimization of the objective function were not achieved. Hybrid particle swarm optimization (PSO) and tabu search (HPSOTS) approach for gene selection for tumor classification were shown in [15]. The incorporation of tabu search (TS) as a local improvement procedure emphasizes the algorithm HPSOTS to overleap restricted optima and demonstrate acceptable performance.

The problem of mining based on heuristic search viewed as a biclustering problem has been lengthily studied in the area of gene expression analysis. The concept of gene expression analysis follows dissimilar formulations of biclustering problems in the circumstance in order to detain various biological associations in the middle of correlated genes and experiment conditions. Classifying trajectories on road networks was not efficient and effective methods for pattern-based classification were discussed in [16].

On the basis of the aforementioned techniques and methods applied, the proposed work uses improved Pearson's correlation proximity-based hierarchical clustering (PCPHC) model to develop biological associations between genes. PCPHC model captures the biological fact that follows a hierarchical clustering model for identifying the biological analysis between genes. To identify the biological association of genes, genes comprising of similar expression patterns are organized using the improved Pearson's correlation proximity model. An efficient pattern growing method GL-PCPHC adopts Seed Augment algorithm which uses average linkage method on rows and columns in order to mine sufficient patterns by growing seed into maximal level.

The PCPHC model makes use of a Seed Augment framework and adopts an average linkage method to mine patterns. Experiments using both synthetic and genuine datasets confirm that the PCPHC model facilitates higher level of quality and also the mining method is considerably efficient. Empirical studies show that the adoption of the PCPHC model improves the quality of the mined patterns compared to the significantly more efficient state-of-the-art heuristic search when it is used to mine strict patterns. The contribution of mining biological association between genes using improved Pearson's correlation proximity-based hierarchical clustering (PCPHC) for gene expression data analysis includes the following:

1. to discover the significant biological association between the genes using improved Pearson's correlation proximity-based hierarchical clustering,
2. to exhaustively mine the gene data analysis using similarity diversion analysis,
3. to adopt global PCPHC model to mine GL-PCPHC patterns,
4. to expand maximal GL-PCPHC pattern that adapts average linkage methods on rows and columns using Seed Augment algorithm.

The rest of the paper is structured as follows.  Section 2 describes the improved Pearson's correlation proximity-based hierarchical clustering model based on the matrices model based on the gene expression data.  Section 3 presents the effective results on the simulation parameter to attain the significance level.  Section 4 evaluated the performance with the table values and graph form. The final section provides the valuable solution with improved quality of the associated patterns.

## 2. Method

### 2.1. Improved Pearson's Correlation Proximity-Based Hierarchical Clustering Model

**Figure 1:**
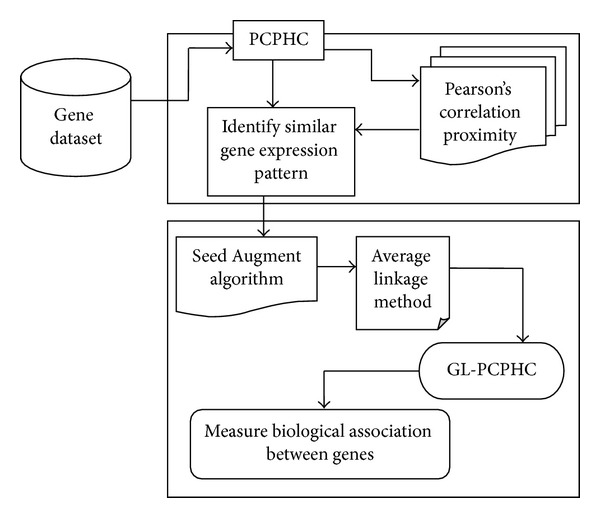
Framework of improved Pearson's correlation proximity-based hierarchical clustering.

Mining biological association between genes using improved Pearson's correlation proximity-based hierarchical clustering (PCPHC) for gene expression data analysis is divided into two parts with the illustrative framework of PCPHC as shown in Figure 1.

The first part involved in PCPHC is to apply the hierarchical clustering model to the input gene dataset with the similarity between two genes expression pattern being measured using the improved Pearson's correlation proximity. The second part in PCPHC is to address the pattern growing method by applying Seed Augment algorithm which uses average linkage method on rows and columns to obtain maximal GL-PCPHC model with genes consisting of similar expression patterns being constructed and the biological association between genes being measured.

Figure 1 shows the architecture diagram of PCPHC model. Gene expression data consists of process by which information from a gene is utilized in the separation of an efficient gene product. The PCPHC model uses microarray initially to estimate the expression level of gene. The identification of biological process for the physiological data present in the gene expression datasets is performed using heuristic search. A heuristic search algorithm provides a collection of genes as the candidate's subjective genes and a division of samples as candidates of gene expression datasets.

### 2.2. Preliminaries

The representation of gene expression data is in the form of an expression matrix. The expression matrix consists of rows “*R*” and columns “*C*” with the intersection of rows and columns being a log ratio factor. Each row denotes the expression of a gene across all experiments whereas each column representing the gene expression levels from a single experiment with the intersection is denoted by a log ratio factor. The log ratio factor for gene expression data is defined as log_2_ (*C*/*R*), where *C* is the gene expression level observed for the testing sample of gene dataset and *R* is the gene expression level observed for the training sample. A gene expression dataset from a microarray experiment is symbolized by a real-valued expression matrix:

(1) $$\begin{array}{c} \text{EM}=\left\{RCw_{i,j}\text{,}\;1\leq i\leq n,\;i\leq j\right\}, \end{array}$$

where the rows *R* = {*r*_1_,…, *r*_*n*_} form the expression patterns of genes and the columns *C* = {*c*_1_,…, *c*_*n*_} represent the expression profiles of samples with log ratio factor denoted by *w*_*ij*_ being the measured expression level of gene *i* in sample *j*. The similarity diversion analysis is present with a novel GL-PCPHC model. The PCPHC model requires that all the genes in a PCPHC model should support the pattern growing method, in the sense that the condition values of a gene in different genes should maintain the ordering relationship between the genes with the conditional values in the similar gene being similar enough. Seed Augment algorithm performs the biological association between the genes.

An efficient and improved Pearson's correlation proximity mines the PCPHC patterns and adopts the average linkage method on rows and columns to mine PCPHC patterns. Then, a new GL-PCPHC mining method using Seed Augment algorithm extracts the PCPHC patterns as seeds and expands them into maximal GL-PCPHC.

### 2.3. Identification of Similar Gene Expression Pattern

Due to the significant characteristics of gene expression data, gene-based clustering presents several new challenges. With the application of improved Pearson's correlation proximity-based heuristic clustering, the challenges are addressed using twofold.

First, with the introduction of gene-based hierarchical clustering algorithm, minimum dependence on prior knowledge is required, which usually is not available before the cluster analysis using gene expression data. Second, due to the experimental complexity involved in the expression matrix, gene expression data comprises a huge amount of noise. Therefore, with the application of improved Pearson's correlation proximity-based heuristic clustering for gene expression data, it significantly extracts relevant information by removing certain level of noise.

Gene-based hierarchical clustering for gene expression dataset produces a hierarchical series of clusters which is illustrated by a tree, called dendrogram. The leaves of dendrogram for gene-based hierarchical clustering not only generate the format ion of the clusters but also record the similarity between the clusters. By removing the dendrogram at certain level, a specific number of clusters for gene dataset are obtained. In improved Pearson's correlation proximity-based hierarchical clustering, each log ratio factor of the gene expression matrix is colored on the basis of the ratio of fluorescence measure whereas the rows of the gene expression matrix are reordered on the basis of the hierarchical dendrogram structure with the help of a constant node-ordering. Once the cluster is being formed, the original gene expression matrix is transformed into a colored table, where higher patches of color denote the genes sharing similar expression patterns.

Given an expression matrix EM = (*R*, *C*) with *R* representing rows and *C* denoting the columns, the methodology followed to mine PCPHC patterns is carried out as follows. The gene expression datasets are represented as vectors as given below:

(2) $$\begin{array}{c} V_{i}=\left\{v_{i,j}\;|\;\text{where}\;j\;\text{lies between}\;1\;\text{and}\;f\right\}, \end{array}$$

where *v*_*i*,*j*_ represents the value of *j*th feature *f* for the *i*th gene value and *f* denotes the features. The proximity level between the two gene values *v*_*i*_ and *v*_*j*_ is obtained with the corresponding vectors *v*_*i*_′ and *v*_*j*_′ using the improved Pearson's coefficient for a measurable dimension dim, where dim *i* = 1,2,…, *n* is given below:

(3) $$\begin{array}{c} \Sigma =\frac{\;1\left(v_{i\dim}-\mu_{vi}\right)\left(v_{j\dim}-\mu_{vj}\right)}{\surd v_{i\dim}-\mu_{vi}\surd v_{j\dim}-\mu_{vj}}, \end{array}$$

where *μ*_*vi*_ and *μ*_*vj*_ are the average values of two vectors *v*_*i*_′ and *v*_*j*_′, respectively.

The proximity level between two gene values using improved Pearson's correlation views each value of gene as a dynamic value with *n* observations and measures the similarity between two genes by evaluating the exponential relationship between the distributions of the two dynamic gene variables. Using the hierarchical clustering algorithm to measure the similar gene expression pattern using the proximity, Pearson's correlation is given above (in Algorithm 1).

### 2.4. Biological Association between Genes

The second part involved in PCPHC is to measure the biological association between genes using the Seed Augment algorithm. Moreover, the Seed Augment algorithm applies average linkage method on rows and columns to expand seed PHC model into GL-PCPHC model and to identify association between the clusters. This as a result helps in the efficient measure of biological association between the genes. The distance between the two-gene expression data is defined as the mean distance between all genes of one group with all the genes of another group:

(4) $$\begin{array}{c} \text{Dist}\left(\text{Gen}1,\text{Gen}2\right)=\frac{1}{\left(N_{\text{Gen}1}*N_{\text{Gen}2}\right)\left(\sum \sum \mathrm{dist}\left(v_{i},v_{j}\right)\right)}, \end{array}$$

where *v*_*i*_ ∈ Gen1 and *v*_*j*_ ∈ Gen2, *i* = 1, 2,…, *N*_Gen1_ and *j* = 1, 2,…, *N*_Gen2_.

Algorithm 2 expands a seed PCPHC by rows and columns which takes the best possible column to associate the current pattern. The row expansion procedure scans the remaining rows that are not incorporated in the current pattern and associates the pattern by those rows that support the best order. Procedures in different orders lead to different pattern association strategies. In order to generate effective association, the column-centric strategy and the row-centric strategy are adopted in GL-PCPHC. The Seed Augment algorithm works with respect to the size of the expression matrix size. As the average linkage method associates with the PCPHC patterns, the thresholds values are set to *e*_max⁡_ and *h*_min⁡_ to be both 0 for fair comparison, which means that the Seed Augment algorithm also mines PCPHC patterns.

Empirical studies show that the application of the PCPHC model improves the quality of the associated patterns. The Seed Augment algorithm that produces GL-PCPHC model is also significantly more efficient than the state-of-the-art PCPHC mining method that proves the robustness of the Seed Augment algorithm.

## 3. Results and Discussion on Gene Expression Data Analysis

The efficiency of the method is evaluated with various conditions using JAVA platform. Initially, the biological process on gene expression data is presented to estimate the biological association performance using yeast gene expression datasets derived from UCI repository and GenBank sequence database. The GenBank sequence database contains the nucleotide sequences and their protein translations produced and maintained by the National Center for Biotechnology Information (NCBI) obtained from the URL ftp://ftp.ncbi.nih.gov/genbank/, for experimental evaluation of the proposed PCPHC model with existing mining discriminative patterns for classifying trajectories, which extract the hidden and practical information from datasets.

The yeast gene expression datasets consist of 8 attributes and 1484 instances with classification associated tasks. The attributes used here for the evaluation of gene expression datasets are Sequence Name (accession number for the SWISS-PROT database), mcg (McGeoch's method for signal sequence recognition), gvh (von Heijne's method for signal sequence recognition), alm (score of the ALOM membrane spanning region prediction program), mit (score of discriminate analysis of the amino acid content of the N-terminal region (20-residue long) of mitochondrial and nonmitochondrial proteins), erl (presence of “HDEL” substring (thought to act as a signal for retention in the endoplasmic reticulum lumen)), binary attribute, pox (peroxisomal targeting signal in the C-terminus), vac (score of discriminate analysis of the amino acid content of vacuolar and extracellular proteins), and nuc (score of discriminate analysis of nuclear localization signals of nuclear and nonnuclear proteins).

The GenBank database is comprised of data from large-scale projects (quality scores) quality score information and consists of integers ranging from 0 to 100; individual files (daily-nc) for new or updated sequence entries, where “MM” represents month and “DD” represents year; data files (wgs) for the sequence-overlap contigs of all whole genome shotgun (livelists), which contains lists, generated weekly on Sunday evening at approximately 6:00 pm EST/EDT, of all nucleotide and protein accession numbers for the sequences in GenBank; and so on.

At first, the biological processes are observed and analyzed for processing. The improved Pearson's correlation proximity-based hierarchical clustering (PCPHC) model is used with the analysis of biological process association and the performance of the PCPHC is measured in terms of execution time, biological association efficiency, pattern quality level, and accuracy rate and gene expression level. Execution time is the average amount of time consumed to perform the whole process in PCPHC model and mechanism with the existing mining discriminative patterns for classifying trajectories (MDP) and triple spectral clustering-based consensus clustering (SC3), measured in terms of milliseconds (ms).

Biological association efficiency is an effective way of biological features associated together in PCPHC and MDP, SC3 measured in terms of percentage (%). Pattern quality level is defined as the amount of accuracy in pattern mining based on the gene expressions and is measured in terms of score points. Accuracy rate is judged by comparing numerous capacities from the same or different sources in gene expression data to attain improved percentage:

(5) Accuracy =Number of correctly associated biological informationTotal number of gene levels.

Gene expression data at each data point designed a DNA microarray that represents the ratio of expression levels of an exacting gene under different experimental conditions. Gene expression level is measured based on the gene count taken for experimental purpose.

### 3.1. Performance of PCPHC Model

The improved Pearson's correlation proximity-based hierarchical clustering (PCPHC) model is compared with the mining discriminative patterns for classifying trajectories (MDP) method and triple spectral clustering-based consensus clustering (SC3).  Table 1 shows the experimental values and graph illustrates the pictorial form of PCPHC model and existing MDP method and SC3 model on various statistical parameters.

Table 1 describes the execution time on PCPHC model and MDP method and SC3 model based on the size of the pattern, where the size of the pattern is measured in terms of kilobytes (KB). As the pattern size increases, execution time also increases, but when compared to the existing MDP and SC3, the execution time decreased gradually. The size of pattern varies on different genes of data.

**Figure 2:**
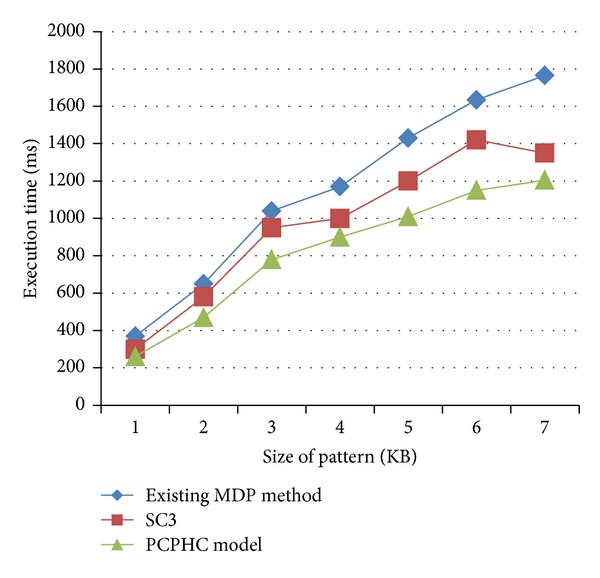
Measure of execution time (using GenBank database).

Figure 2 describes the execution time based on the size of the pattern processed using GenBank database. The execution time of the PCPHC model is 30–42% less when compared with the MDP method and SC3 model, because all the genes in a PCPHC pattern support a distance based on the proximity using Pearson's correlation in the sense that the condition values of a gene maintain the ordering relationship between all the genes datasets to reduce the time. But comparatively, SC3 is better than the MDP method because SC3 uses spectral clustering for cancer discovery from gene expression profiles resulting in better execution time. As the execution time decreased, the processing speed in PCPHC improved.

Table 2 describes the biological association efficiency based on the feature set using GenBank database. As the features increased, biological association efficiency percentage also improved drastically in PCPHC model.

**Figure 3:**
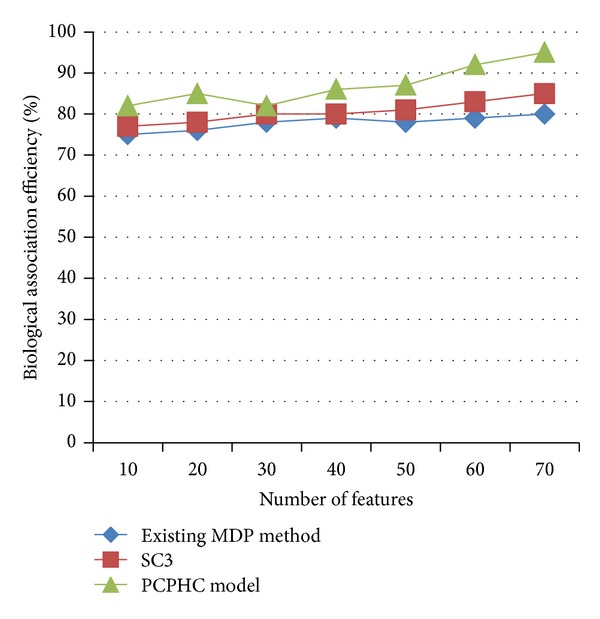
Measure of biological association efficiency (using yeast gene expression dataset).

Figure 3 describes the biological association efficiency based on the number of features. The PCPHC biological association is 4–15% improved compared to the MPD and 2–10% improved compared to the existing SC3, as the GL-PCPHC average linkage method on rows and columns in order to capture the underlying consensus trend and support in such a way that the similarities between the gene expression patterns and between the clusters are large enough.

**Figure 4:**
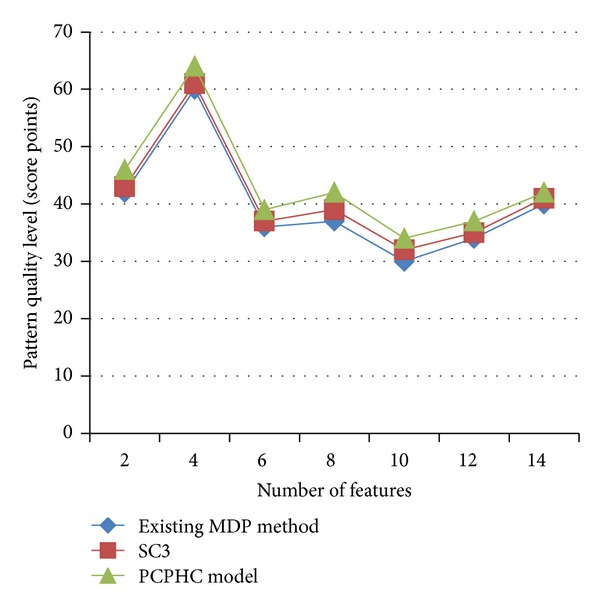
Measure of pattern quality level.

Figure 4 and Table 3 describe the pattern quality level based on the significance level obtained using the yeast gene expression dataset. Significance level ranges from 2,4,… to 14 on the gene expression data. As the significance level increases, pattern quality level improves from 6 to 11% due to the algorithm development in PCPHC model and 2–6% more than the existing MDP method and SC3 model, respectively. PCPHC adopts the proximity measure based on the improved Pearson's correlation which always takes the best possible log ratio factor to associate the current pattern and improve the pattern level effectively.

Procedures in different orders lead to different pattern association strategies in PCPHC model when compared with the MDP method and SC3 model.

**Figure 5:**
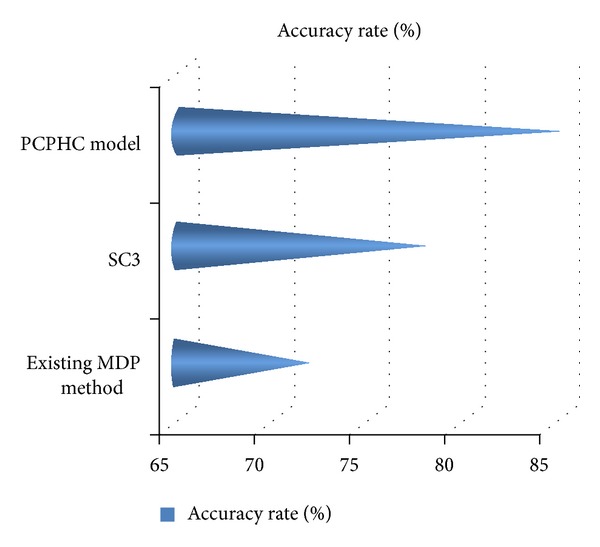
Technique versus accuracy rate (using GenBank database).

Figure 5 and Table 4 show the accuracy rate of existing MDP method and SC3 model and are compared against the PCPHC model in terms of percentage (%) using GenBank database. Linear prefix tree (LPTree) structure organizes the candidate linear orders and improves the accuracy rate to 15% when compared with the MDP method and 8% better than the SC3 model. The accuracy rate using SC3 model is better than the MDP method because the SC3 model used separately a consensus function to split the consensus matrix constructed from multiple clustering solutions resulting in increased accuracy rate. The Seed Augment algorithm rapidly updated candidate using the column expansion and row expansion function. Performing the expansion helps generate the candidate linear orders and count the supports effectively in PCPHC model.

Table 5 describes gene expression level based on the genes. As the gene data increases, gene expression level is improved drastically and illustrated in terms of percentage (%).

**Figure 6:**
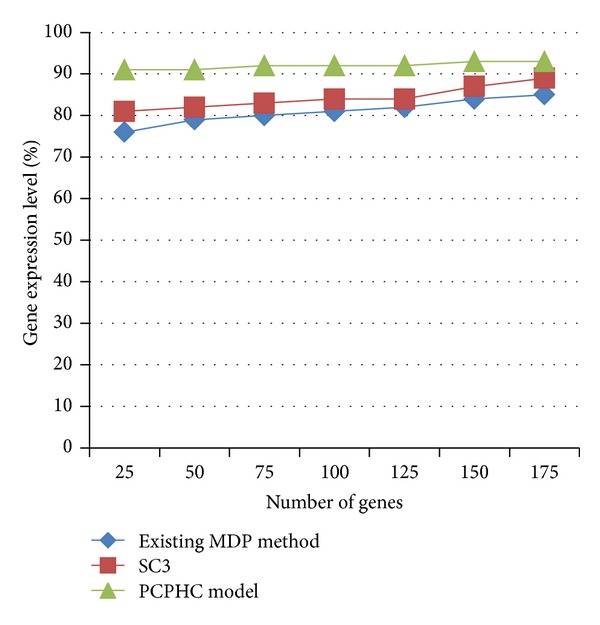
Measure of gene expression level (using yeast gene expression dataset).

Figure 6 describes the gene expression level based on the PCPHC model and compared with the MDP method and SC3 model. Gene expression level in PCPHC model is 8–16% increased because of proximity based on the improved Pearson's correlation method that shares the analogous principle with the sequential pattern mining method. Gene ranges from 25,50,…, 175. In a similar manner, when compared to the MDP method, the gene expression level was improved using SC3 model ranging from 4 to 10% because it also reduces the effect of noisy genes in cancer gene expression profiles.

Finally, it is being observed that the improved Pearson's correlation proximity-based hierarchical clustering (PCPHC) model develops biological associations between genes and correlated genes identified as a linear order. The PCPH model requires that the rows in a PCPHC support Seed Augment framework and adopts an improved Pearson's correlation proximity-based model to mine patterns. Empirical studies show that the adoption of the PCPHC model improves the quality of the mined patterns.

## 4. Conclusion

The improved Pearson's correlation proximity-based hierarchical clustering (PCPHC) model is an efficient model which exhaustively mine the similarity diversion strategy interpretively. A global PCPHC (GL-PCPHC) model adopts pattern growing method to mine GL-PCPHC patterns and discover significant biological associations between genes. PCPHC model allows linear orders and Seed Augment algorithm adopts two different growing strategies on rows and columns in order to expand a seed PCPHC model into a maximal GL-PCPHC pattern. The high rate of expression levels between gene datasets is also simultaneously measured using the improved Pearson's correlation. Experimental studies show that the PCPHC model outperforms all the current models, and, importantly, it leads to the discovery of more quality patterns. The experimental result of PCPHC model attains the improved gene expressional data, minimal execution time, 10. 085% effective biological association based on feature set, 4.5% maximal accuracy rate, and better pattern quality level based on the significance level.

## Figures and Tables

**Algorithm 1:**
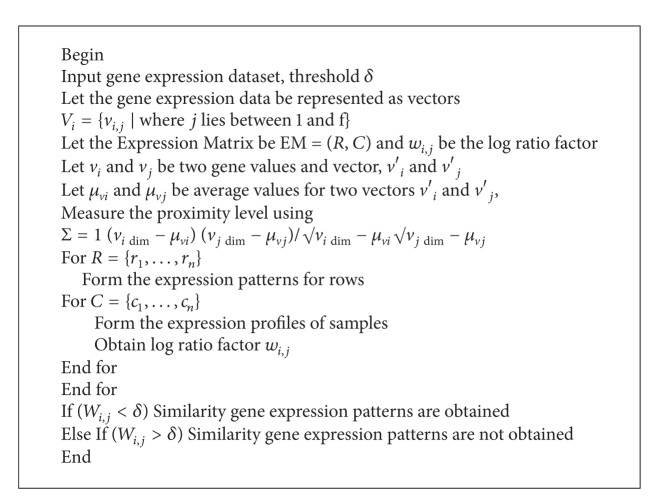
Hierarchical clustering algorithm to measure the similar gene expression pattern.

**Algorithm 2:**
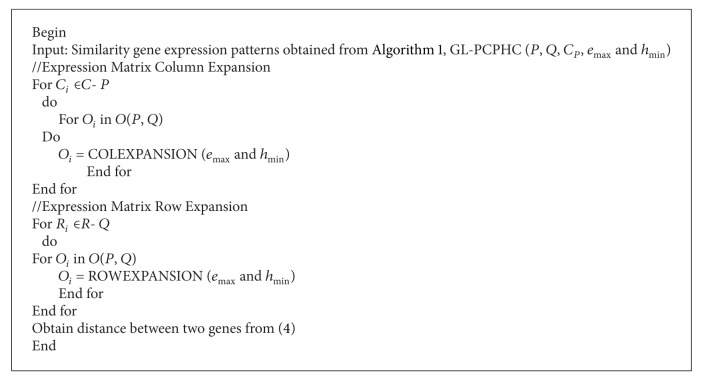
Seed Augment algorithm.

**Table 1:** Tabulation of execution time (using GenBank database).

Size of pattern (KB)	Execution time (ms)		
	Existing MDP method	SC3	PCPHC model
155	370	300	260
203	650	580	470
350	1040	950	780
489	1170	1000	900
550	1430	1200	1010
614	1635	1420	1150
757	1765	1350	1205

**Table 2:** Tabulation of biological association efficiency (using yeast gene expression dataset).

Number of features	Biological association efficiency (%)		
	Existing MDP method	SC3	PCPHC model
10	75	77	82
20	76	78	85
30	78	80	82
40	79	80	86
50	78	81	87
60	79	83	92
70	80	85	95

**Table 3:** Tabulation of pattern quality level.

Number of features	Pattern quality level (score points)		
	Existing MDP method	SC3	PCPHC model
2	42	43	46
4	60	61	64
6	36	37	39
8	37	39	42
10	30	32	34
12	34	35	37
14	40	41	42

**Table 4:** Technique versus accuracy rate (using GenBank database).

Technique	Accuracy rate (%)
Existing MDP method	72
SC3	78
PCPHC model	85

**Table 5:** Tabulation of gene expression level (using yeast gene expression dataset).

Number of genes	Gene expression level (%)		
	Existing MDP method	SC3	PCPHC model
25	76	81	91
50	79	82	91
75	80	83	92
100	81	84	92
125	82	84	92
150	84	87	93
175	85	89	93
